# Effect of a Novel Pocket Compression Device on Hematomas Following Cardiac Electronic Device Implantation in Patients Receiving Direct Oral Anticoagulants

**DOI:** 10.3389/fcvm.2022.817453

**Published:** 2022-02-24

**Authors:** Ye-Ping Fei, Lei Wang, Chun-Yan Zhu, Jing-Chao Sun, Hui-Lin Hu, Chang-Lin Zhai, Chao-Jie He

**Affiliations:** ^1^Department of Cardiology, The Affiliated Hospital of Jiaxing University, Jiaxing, China; ^2^Department of General Practice, The Affiliated Hospital of Jiaxing University, Jiaxing, China

**Keywords:** cardiovascular electronic implantable device, pocket compression device, pocket hematoma, direct oral anticoagulants, skin erosion

## Abstract

**Background:**

A pocket hematoma is a well-recognized complication that occurs after pacemaker or defibrillator implantation. It is associated with increased pocket infection and hospital stay. Patients suffering from atrial fibrillation and undergoing cardiovascular electronic implantable device (CIED) surgery are widely prescribed and treated with direct oral anticoagulants (DOACs). In this study, the use of a novel compression device was evaluated to examine its ability to decrease the incidence of pocket hematomas following device implantation with uninterrupted DOACs.

**Methods:**

A total of 204 participants who received DOACs and underwent CIED implantation were randomized into an experimental group (novel compression device) and a control group (elastic adhesive tape with a sandbag). The primary outcome was pocket hematoma, and the secondary outcomes were skin erosions and patient comfort score. Grade 3 hematoma was defined as a hematoma that required anticoagulation therapy interruption, re-operation, or prolonged hospital stay.

**Results:**

The baseline characteristics of both groups had no significant differences. The incidence of grades 1 and 2 hematomas was significantly lower in the compression device group than in the conventional pressure dressing group (7.8 vs. 23.5 and 2.0 vs. 5.9%, respectively; *P* < 0.01). Grade 3 hematoma occurred in 2 of 102 patients in the experimental group and 7 of 102 patients in the control group (2.0 vs. 6.9%; *P* = 0.03). The incidence rates of skin erosion were significantly lower, and the patient comfort score was much higher in the compression device group than in the control group (*P* < 0.01). Multivariable logistic regression analysis showed that the use of novel compression device was a significant protective factor for pocket hematoma (OR = 0.42; 95% CI, 0.29–0.69, *P* = 0.01).

**Conclusions:**

The incidence of pocket hematomas and skin erosions significantly decreases when the proposed compression device is used for patients undergoing device implantation with uninterrupted DOACs. Thus, the length of hospital stay and re-operation rate can be reduced, and patient comfort can be improved.

**Clinical Trial Registration:**

http://www.chictr.org.cn, identifier: ChiCTR2100049430.

## Introduction

Cardiovascular electronic implantable devices (CIEDs), such as implantable pacemakers, implantable cardioverter–defibrillators (ICDs), and cardiac resynchronization therapy defibrillators, have become the standard therapy utilized in the management of different cardiac conditions (e.g., bradyarrhythmias), primary and secondary preventions against sudden cardiac death, and amelioration of congestive heart failure (1–3). With a consistent increase in human life expectancy and advancement in medical technology, approximately 1.2–1.4 million CIEDs are implanted each year globally (4). However, CIED procedures may cause complications. A well-recognized complication associated with these procedures is pocket hematoma, which is reported in 2–9% of patients. Patients on antithrombotic therapy have an increased risk of having pocket hematoma (5–7).

Antithrombotic management in patients undergoing CIED implantation and requiring long-term oral anticoagulants or antiplatelet therapy is a growing strategic dilemma (8). The number of prescribed direct oral anticoagulants (DOACs) has grown substantially since the publication of the BRUISE CONTROL trial. DOACs are widely used to prevent stroke in patients with non-valvular atrial fibrillation (9, 10). However, approximately 25% of patients undergoing CIED implantation require long-term DOACs, leading to the increased incidence rates of perioperative bleeding (11). Furthermore, the temporary withdrawal of these anticoagulant drugs prior to device implantation poses a three-fold increased risk of ischemic stroke, myocardial infarction, and systemic thromboembolism, especially in moderate-to-high-risk patients (12, 13). The continuation of DOACs during the perioperative period also increases the risk of pocket hematoma (14, 15).

Traditionally, a pressure dressing fixed with elastic adhesive tape that is then covered with a sandbag has been used to prevent pocket hematoma after CIED implantation. However, the sandbag often migrates from its fixed position because of the unique location of the device pocket. It does not provide adequate pressure, resulting in the need for repeated fixation and exacerbating patient discomfort (16). Furthermore, adhesive tapes designed for pressure dressings may lead to skin erosions. No commercially available devices have been shown to be effective in patients with uninterrupted DOACs after CIED implantation. Therefore, we developed a novel compression device to reduce the incidence of postoperative pocket hematomas and improve patient comfort. In this randomized study, we aimed to assess the efficacy of the proposed compression device in preventing pocket hematomas compared with that of conventional techniques in patients on uninterrupted DOACs.

## Methods

### Study Population and Design

This single-center randomized controlled clinical trial was conducted in the heart center affiliated with Jiaxing University from July 2020 to May 2021. Approximately 1,000 devices were implanted each year in this center. All the patients admitted for the permanent CIED implantation of a pacemaker, ICD, or CRT-D were considered in this study. They were included if they were ≥18 years old, undergoing CIED implantation, and on long-term DOAC (dabigatran and rivaroxaban) therapy. The exclusion criteria were as follows: (1) without anticoagulation, (2) anticoagulation with warfarin, (3) history of any psychiatric illness, (4) coagulation disorder or severe anemia, and (5) refusal to participate. Patients with a body mass index of >35 kg/m^2^ were also excluded from this trial because the novel compression device was designed for individuals with normal body sizes. A total of 204 participants screened for eligibility were randomized at 1:1 into an experimental group and a control group by using a random number table and stratified by device type. Patients in both groups were instructed to continue taking aspirin, clopidogrel, or ticagrelor because of high-risk clinical features at their cardiologist's discretion.

This study was approved by the Human Ethics Research Committee of the Affiliated Hospital of Jiaxing University. All the participants were asked to sign their informed consent before enrollment. The trial was registered at the Chinese Clinical Trial Registry (ChiCTR; http://www.chictr.org.cn; ChiCTR2100049430).

### Device Implantation and Postprocedure Management

All procedures were performed by a team of experienced cardiologists, who each had experience of at least 300 device implantations. After prophylactic antibiotics (first-generation cephalosporin or macrolides to patients with a penicillin allergy) and local anesthesia were administered, a pectoral incision was made. Venous access was obtained by puncturing the subclavian vein, and leads were implanted under fluoroscopic guidance in a cath lab. Active fixation was applied to all of the atrial leads and the majority of the ventricular leads. All devices were implanted without the use of electrocautery, and the wound was closed with absorbable suture.

The patients were advised to remain in a supine position for 8 h during the immediate postoperative period. Then, they were gradually moved from a 30° semireclining position to a sitting position. They were encouraged to get out of bed and walk around 24 h after their operation, but they were instructed to avoid extending their ipsilateral shoulder during physical activities. Standard device interrogation and chest radiograph were conducted at an appropriate time after CIED implantation. The pocket was assessed on postoperative days 1, 3, and 7.

### Novel Compression Device and Conventional Pressure Dressing

**Figure 1:**
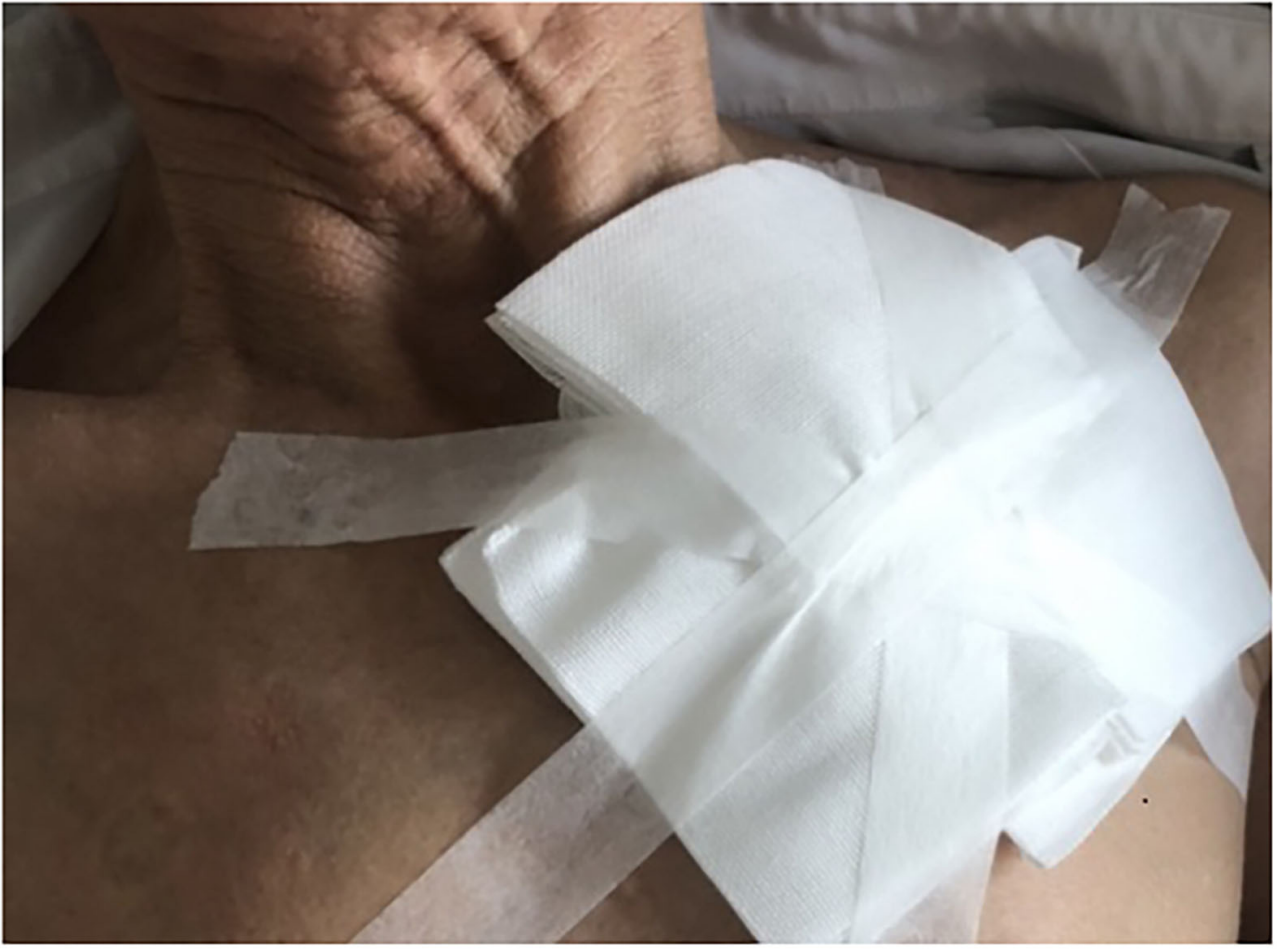
Conventional pressure dressing with adhesive tape.

**Figure 2:**
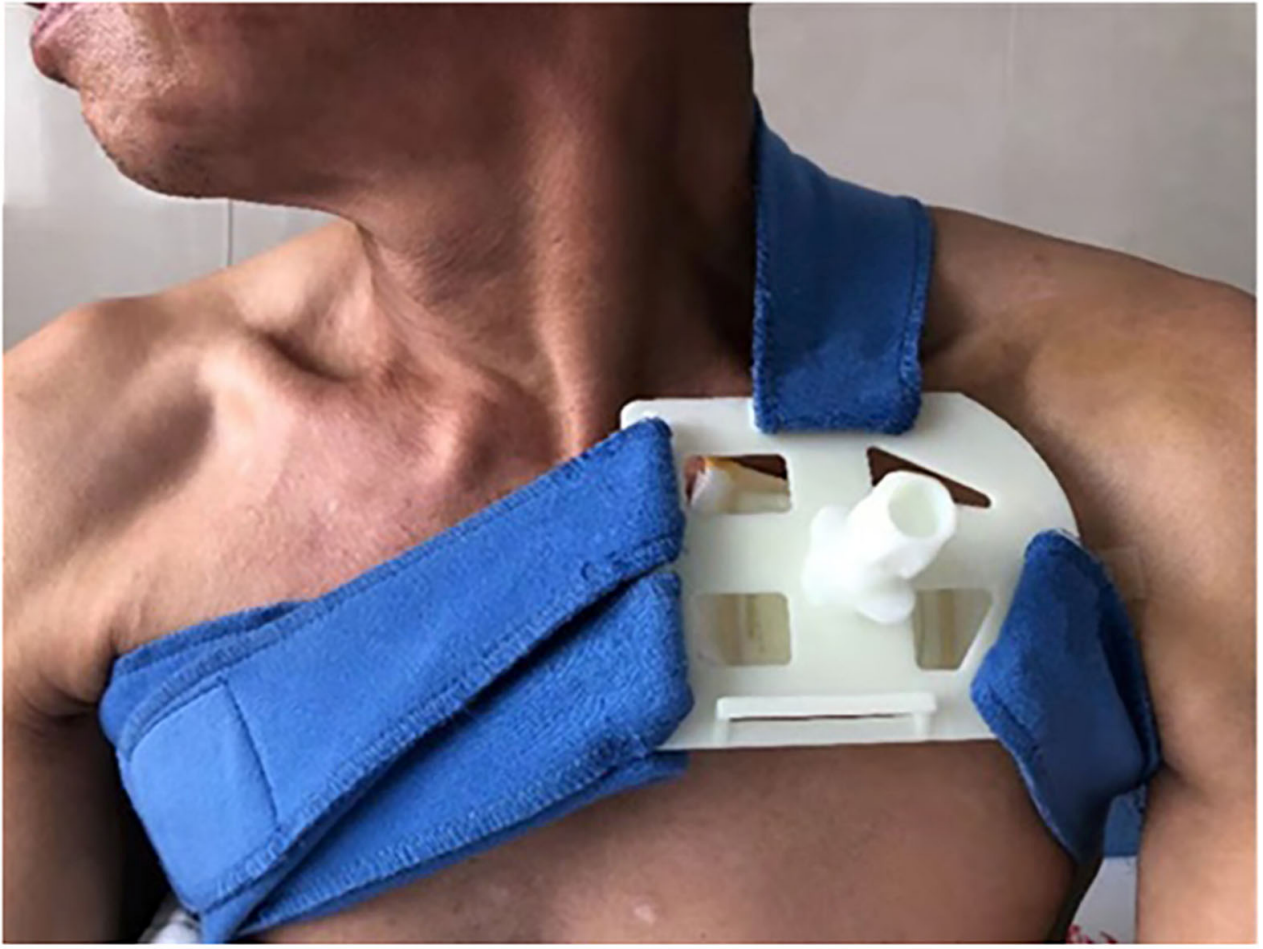
Novel pocket compression device.

**Figure 3:**
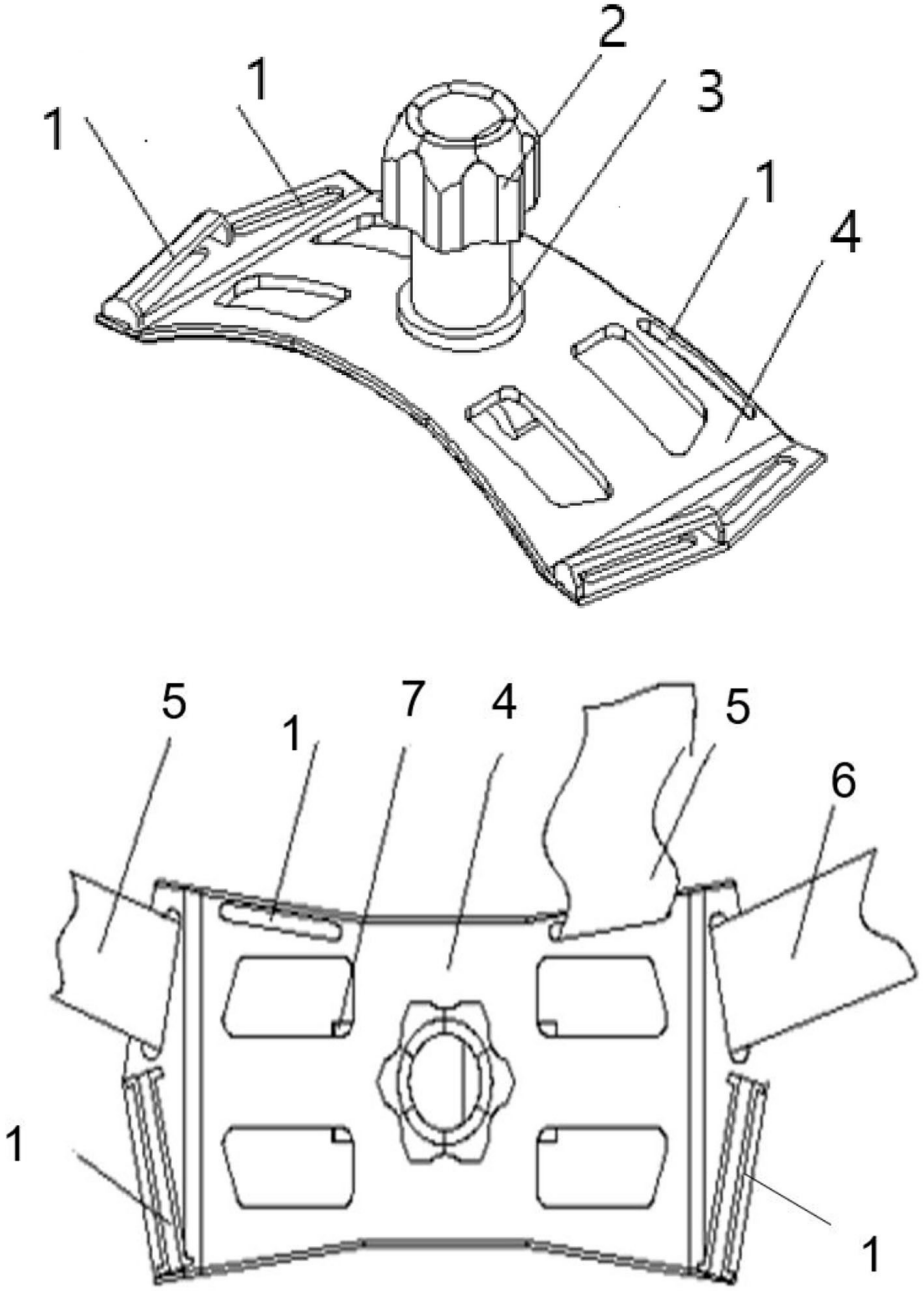
Structural diagram of the new compression device. 1, Shoulder strap fixing hole; 2, pressure-adjusting knob; 3, pressure-adjusting screw; 4, support plate; 5, shoulder band; 6, chest band; and 7, breather hole.

All the participants were asked to stay in bed for 24 h after CIED implantation. A conventional pressure dressing or the novel compression device was employed immediately, and vital signs were monitored. Conventional pressure dressing, which is commonly used, was applied to cover the surgical incision. The gauze was kept in place with adhesive tape, and the sandbag was adjusted repeatedly to maintain its location for adequate pressure and ensure the patient's comfort (Figure 1). Granted with a national patent (Chinese patent number: ZL 2014 1 0037276.7), the self-design novel pocket compression device was composed of a support plate, a shoulder band, a pressure-adjusting knob, and a pressure-adjusting screw. It was constructed from cotton fabric, medical plastic, and silica gel (Figures 2, 3). The pressure-adjusting knob was manually adjusted by medical staff based on previous experience and patient comfort. The novel compression device did not require adhesive tape, which can cause skin erosion. For the novel compression device group, the wound was locally pressurized for 8 h, and the screw was reversely adjusted by two to three turns every 2 h to reduce pressure. In the control group, a traditional sandbag was used after the procedure, placed directly above the dressing, and relaxed for 10 min every 2 h. The sandbags were removed after 8 h.

### Outcome Measures

The primary outcomes were grade 1, 2, and 3 pocket hematomas between the groups after CIED procedures. The grading scale of hematoma types was as follows: grade 1 hematoma if a patient experienced ecchymosis or mild effusion around the surgical incision, no swelling, or pain in the device pocket; grade 2 hematoma if a patient suffered from large effusion in the pocket leading to swelling and causing functional impairment or pain to the device pocket; and grade 3 hematoma, also known as clinically significant hematoma, was defined according to the BRUISE CONTROL trial as any pocket hematoma requiring re-operation, resulting in the prolonged hospital stay (defined as extended hospitalization for >24 h primarily due to hematoma), and requiring interruption or reversal of DOACs (defined as intentional withholding, or an antidote indicated for the reversal of DOACs was used in response to pocket hematoma, resulting in subtherapeutic anticoagulation for >24 hours) [17. The decision for the interruption of anticoagulation therapy or requiring a second operation or prolonged hospital stay was made by two cardiologists who independently evaluated the wound without any information about group assignment.

The secondary outcomes were skin erosions and patient comfort scores. Skin erosions were defined as skin damage observed during the removal of the adhesive tape or dressing (17). The comfort level of the patients was assessed on a visual analog scale (VAS) during the application of a sandbag or compression. For comfort level assessment, the patients were required to describe how they felt on a 100 mm VAS ranging from “0” as “very uncomfortable” to “10” as “very comfortable” (18).

### Statistical Analysis

The sample size calculation was based on our pilot study, a minimum sample of 200 participants were required to have 90 power to detect a 40% relative reduction in incidence of pocket hematoma in compression device group with an α of 0.05. Data were statistically analyzed with SPSS v.23 (SPSS Inc., Chicago, IL, USA). Continuous variables were expressed as means ± SD for continuous variables and percentages for qualitative variables. They were then compared using a *T*-test or a Mann–Whitney U test. Categorical variables were summarized as numbers and percentages and compared via chi-square or Fisher's exact test. Logistic regression model was applied to identify the predictors of pocket hematomas. Age, diabetes, heart failure, packmaker, ICD/CRT-D, aspirin, clopidogrel, procedure duration, and novel compression device were considered as covariates in the univariate analysis. For the multivariate models, clinical risk factors which were univariate predictors (*P* < 0.01) were regarded as covariates. Data with *P* < 0.05 were considered statistically significant.

## Results

### Comparison of Patient Characteristics

**Figure 4:**
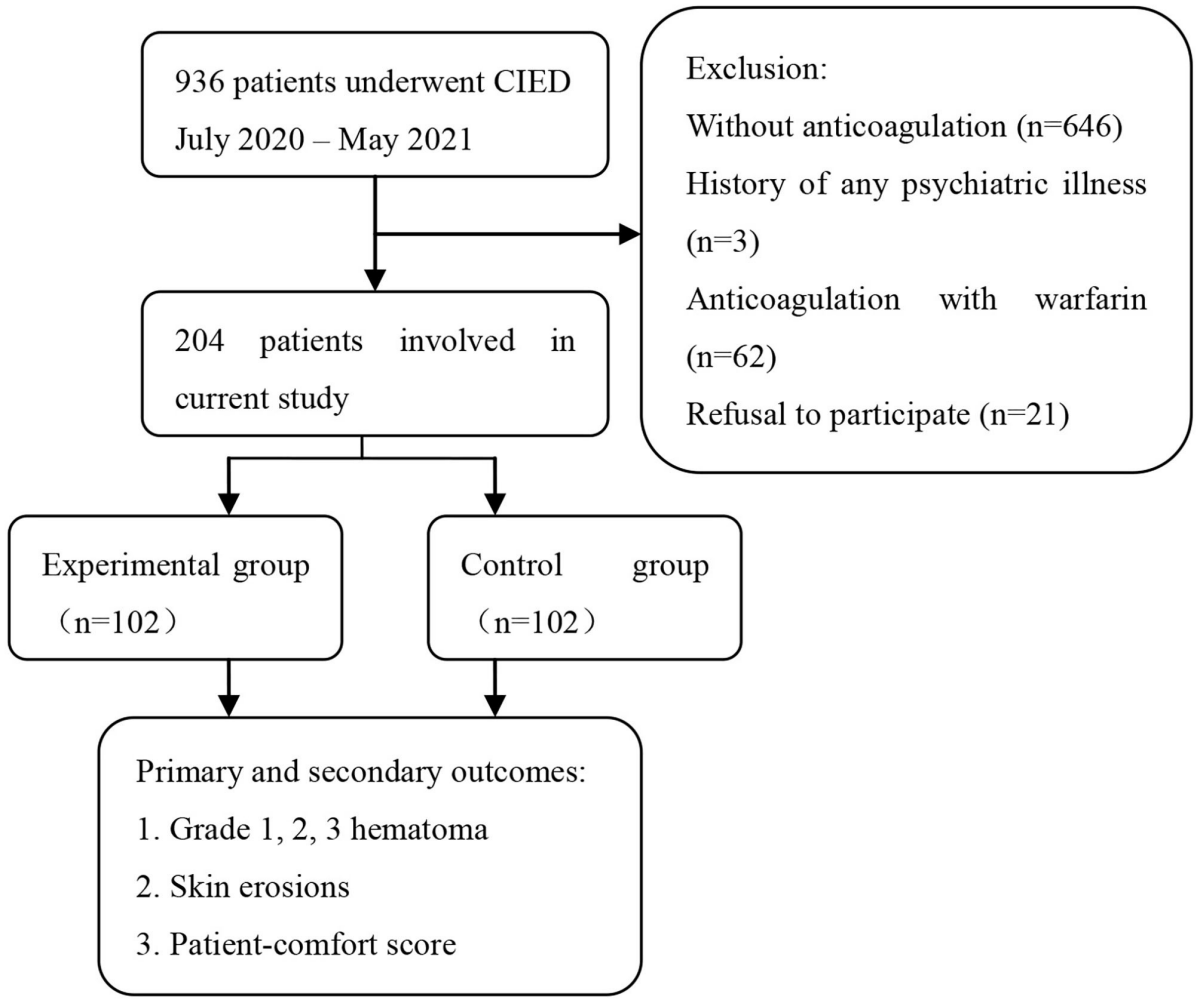
Flowchart of the study.

The demographics and clinical characteristics of the study population are displayed in Table 1. Of the 936 patients included in the study, 204 were recruited because they were on DOAC therapy at the time of device implantation. A total of 732 patients were excluded from the present study because of the following: 646 did not take any anticoagulant therapy, 62 received anticoagulation with warfarin, three had a history of psychiatric illness, and 21 refused to participate in the study (Figure 4).

**Table 1:** Demographic and clinical characteristics of the study population.

**Characteristic**	**Compression device**	**Control (*n* = 102)**	***P*-value**
**Demographics**
Age, mean ± SD, y	73.5 ± 7.4	71.1 ± 7.2	0.04
Male, *n* (%)	66 (64.7)	63 (61.8)	0.36
BMI, mean ± SD, kg/m^2^	23.8 ± 3.1	23.6 ± 3.0	0.77
CHA_2_DS_2_-VASc score	3.7 ± 1.3	3.6 ± 1.3	0.86
**Comorbid condition**, ***n*** **(%)**
Hypertension	59 (57.8)	63 (61.8)	0.47
Diabetes	32 (31.4)	33 (32.4)	0.72
Coronary heart disease	30 (29.4)	32 (31.4)	0.69
Heart failure	32 (31.4)	32 (31.4)	0.99
Sinus node dysfunction	36 (35.3)	33 (32.3)	0.62
Atrioventricular block	47 (46.1)	48 (47.1)	0.86
**Laboratory parameters, mean** **±SD**
PT, s	23.5 ± 1.3	23.8 ± 1.4	0.63
APTT, s	40.9 ± 2.8	41.2 ± 2.9	0.33
Creatinine, umol/L	74.2 ± 13.6	73.6 ± 13.3	0.45
Hemoglobin, g/L	126.2 ± 8.5	128.2 ± 8.7	0.23
Platelet count, 10^∧^9/L	202 ± 55	210 ± 58	0.12
LVEF, %	40 ± 12	41 ± 12	0.86
**Direct oral anticoagulant**, ***n*** **(%)**
Dabigatran	42 (41.2)	44 (43.1)	0.65
Rivaroxaban	60 (58.8)	58 (56.9)	0.63
**Concomitant antiplatelet**
**medication**, ***n*** **(%)**
Aspirin	24 (23.5)	20 (19.6)	0.33
Clopidogrel	16 (15.7)	18 (17.6)	0.42
**Type of device**, ***n*** **(%)**
Pacermaker	59 (57.8)	58 (56.9)	0.69
Implantable cardioverter defibrillator	7 (6.9)	8 (7.8)	0.52
CRT and CRT-D	10 (9.8)	11 (10.8)	0.77
Generator exchange	26 (25.5)	25 (24.5)	0.54
Operation duration, mean ± SD, min	82.2 ± 33.5	85.2 ± 37.0	0.23

*SD, standard deviation; PT, prothrombin time; APTT, activated partial thromboplastin time; LEVF, left ventricular ejection fraction; CRT, cardiac resynchronization therapy; CRT-D, defibrillator cardiac resynchronization therapy*.

The baseline characteristics had no significant differences between the groups, although the patients in the novel compression device group (73.5 ± 7.4 years) were older than those in the control group (71.1 ± 7.2 years, *P* = 0.04). Of the patients on DOAC medication, 86/204 (42.2%) were on dabigatran, and 118/204 (57.8%) were on rivaroxaban. Notably, 70 (34.3%) were concomitant on antiplatelet therapy: aspirin (17.6%), clopidogrel (12.7%), and aspirin plus clopidogrel (3.9%). As expected, both groups had a prolonged prothrombin time (23.5 ± 1.3 and 23.8 ± 1.4) and activated partial thromboplastin time (40.9 ± 2.8 and 41.2 ± 2.9). All the patients underwent CIED procedures consisting of 117 permanent pacemaker implantations, 21 CRT and CRT-D, 15 ICD implantations and 51 generator exchange (Table 1).

### Primary and Secondary Outcomes

The primary outcome of pocket hematomas occurred in 12/102 (11.8%) patients in the experimental group and 37/102 (36.3%) in the control group. Grade 3 hematoma was detected in nine cases, which required the interruption of anticoagulation therapy, re-operation or prolonged hospital stay, grade 2 hematoma was found in eight cases, and grade 1 hematoma was observed in the remaining cases (Table 2). The application of the novel pocket compression device resulted in a statistically significant reduction in the overall incidence of hematomas (11.8 vs. 36.3%, *P* < 0.01). Grades 1, 2, and 3 hematomas were observed in 8, 2, and 2 patients in the compression device group, respectively. In the control group, these hematomas were found in 24, 6, and 7 patients, respectively. The incidence of grades 1, 2, and 3 pocket hematomas significantly decreased in the experimental group compared with that in the control group (7.8 vs. 23.5% [*P* < 0.01], 2.0 vs. 5.9% [*P* < 0.01], 2.0 vs. 6.9% [*P* = 0.03], respectively). In experimental group, there was only 1 case of grade 3 hematoma in CRT-D and generator exchange procedures, respectively. In the control group, the incidence of grade 3 hematoma in pacemaker, generator exchange, CRT-D procedure were 1, 2, and 4, respectively. The incidence of grade 3 hematoma in CRT-D procedure was significantly higher in the compression device group than in the control group (1.0 vs. 3.9%, *P* < 0.01, Table 3). In multivariable analysis demonstrated no significant association between age, diabetes, heart failure, type of CIEDs implanted, antiplatelet agent, or procedure duration and pocket hematoma (Table 4). The use of novel compression device was a significant protective factor for pocket hematoma (OR = 0.42; 95% CI, 0.29–0.69, *P* = 0.01).

**Table 2:** Primary and secondary endpoint of the current study.

**Outcome**	**Novel device (*n* = 102)**	**Control (*n* = 102)**	***P*-value**
**Primary endpoint**, ***n*** **(%)**
Grade 1 hematoma	8 (7.8)	24 (23.5)	<0.01
Grade 2 hematoma	2 (2.0)	6 (5.9)	<0.01
Grade 3 hematoma	2 (2.0)	7 (6.8)	0.03
**Secondary endpoint**
Skin erosions, *n* (%)	0 (0)	9 (8.8)	<0.01
Patient comfort score, mean ± SD	7.2 ± 1.1	4.8± 0.8	<0.01

**Table 3:** The incidence of grade 3 hematoma in different type of devices.

**Type of devices, *n* (%)**	**Compression device (*n* = 102)**	**Control (*n* = 102)**	***P*-value**
Pacemaker	0 (0)	1 (1.0)	0.76
ICD	1 (1.0)	2 (2.0)	0.45
CRT-D	1 (1.0)	4 (3.9)	<0.01

*ICD, implantable cardioverter defibrillator; CRT-D, defibrillator cardiac resynchronization therapy*.

**Table 4:** Univariate and multivariate analysis for predictors of pocket hematoma.

	**Univariate analysis**		**Multivariable analysis**	
**Variables**	**OR (95% CI)**	***P*-value**	**OR (95% CI)**	***P*-value**
Age	1.03 (0.87–1.83)	0.63	NA	NA
Diabetes	1.19 (0.96–3.98)	0.09	1.21 (0.97–4.11)	0.07
Heart failure	1.11 (0.74–3.75)	0.36	NA	NA
Packmaker	0.98 (0.94–1.12)	0.37	NA	NA
ICD and CRT-D	1.69 (1.26–2.74)	0.03	1.64 (1.21–2.70)	0.02
Aspirin	1.24 (0.95–1.49)	0.08	1.17 (0.91–1.24)	0.11
Clopidogrel	1.17 (0.92–1.39)	0.16	NA	NA
Procedure duration	1.10 (0.89–2.74)	0.86	NA	NA
Novel compression device	0.45 (0.33–0.74)	0.02	0.42 (0.29–0.69)	0.01

*OR, odds ratio; CI, confidence interval; NA, not applicable; ICD, implantable cardioverter defibrillator; CRT-D, defibrillator cardiac resynchronization therapy*.

For secondary outcomes, none of the participants in the novel compression device group experienced skin erosion, but 9 of the participants in the control group had skin erosion (0 vs. 8.8%, *P* < 0.01). The patient comfort score in the experimental group was significantly higher than that in the control group (7.2 ± 1.1 vs. 4.8 ± 0.8, *P* < 0.01).

## Discussion

Pocket hematoma is a major complication after CIED implantations, and it increases the risk of device-related infections (6, 19). In this prospective randomized trial, the efficacy of the proposed novel compression device was evaluated and compared with conventional techniques in patients undergoing electronic device implantations. We found that our self-design pocket compression device was associated with a significantly lower rate of pocket hematoma and skin erosions than that of traditional methods. It could also simultaneously improve patient comfort compared with traditional techniques involving elastic adhesive tape and sandbags. These results also suggested that continued DOAC strategy might be reasonable for patients during the perioperative period. Our results were consistent with the findings of some small cohort studies that continued DOACs during device surgery (20, 21).

Several studies have assessed the occurrence of pocket hematoma, from 0.9% to 28.7%. (16, 22–26). In BRUISE CONTROL trial, the clinically significant device-pocket hematoma occurred in 12 of 343 patients (3.5%) in the continued-warfarin group, as compared with 54 of 338 (16.0%) in the heparin bridging group (27). The frequency of grade 3 hematoma in this study (4.4%) was higher than the 3.5% reported in the BRUISE CONTROL trial despite compression devices and the exclusion of warfarin users. Different anticoagulants used in the trials, usage of electrocautery, and difference of investigator adjudication on pocket hematoma maybe the main interpretations to this discrepancy. The incidence of hematoma in Hu et al.'s study was 13.0 and 44.4% in experimental group and control group (16). In the present study, the incidence of hematoma was 24.0% upon discharge, while the incidence of grade 3 hematoma was only 4.4%. This discrepancy could be attributed to some factors, including differences in study design, baseline characteristics of patients, inconsistencies in the definition of pocket hematoma and non-use of electrocautery (9, 22). In our trial, electrocautery was not employed since the operators do not use electrocautery routinely and the hematoma was defined in accordance with the classification of De Sensi et al. who categorized pocket hematomas into three grades based on clinical phenomena and required interventions. All the clinical findings around the pocket and those leading to therapeutic interventions were recorded in our study, whereas a number of previous studies only included clinically significant hematomas equivalent to grade 3 hematoma in our trial (17, 22, 26).

Prospective studies have been conducted to explore the application of pocket compression after procedures to prevent hematomas (16, 22, 23, 28). For example, Awada et al. performed a prospective registry study and showed that the use of a marketed compression device approved in Germany can significantly reduce the incidence of pocket hematomas and subsequent infections compared with the control device in patients receiving anticoagulation or dual antiplatelet therapy and undergoing CIED implantation (23). Similarly, another study evaluated a postsurgical vest among 40 patients with the interruption of warfarin and antiplatelet agents during procedures and demonstrated a significant reduction in the occurrence of pocket hematomas (28). Notably, these studies included only the patients on therapeutic warfarin and excluded those receiving DOACs. Therefore, their results might not be applicable to patients on DOACs. Hu et al. conducted a randomized controlled trial involving 108 patients and revealed that a pocket compression device can significantly reduce the occurrence of hematomas and adverse skin reactions after device implantation (16). However, only a small proportion of patients continued antithrombotic medication in their trial. Furthermore, our novel compression tool has a great advantage in terms of clinical practical use since none of their compression devices have a pressure regulator function.

DOACs are easier to administer and obviate the need for regular international normalized ratio monitoring required for warfarin therapy. The number of patients treated with DOACs has increased significantly in clinical practice (29). Hence, there is also an increased risk of bleeding complications especially in patients on multiple antithrombotic agents such as coronary heart disease or receiving valve surgery (30). To our knowledge, this research was the first prospective cohort study with a relatively large sample to evaluate the use of a novel pocket compression device for decreasing the incidence of pocket hematomas following CIED implantation with uninterrupted DOACs. Some studies have indicated various hematoma predisposing procedural and patient factors, including aging, antiplatelet usage, heart failure, and device implantation type (26, 31). Our findings showed that antiplatelet drugs, device implantation type, and pocket hematoma incidence had no significant association, possibly because of the small sample size from one heart center.

The proposed novel pocket compression device has several advantages over the use of elastic adhesive tape and sandbags. First, the specially designed shoulder band and chest band can certainly fix the device over the pocket and sustain the pressure on it when patients change their body position. Second, the pressure-adjusting knob and screw can be manually adjusted to control the tightness of the compression device based on the physician's experience and the patient's comfort while applying the appropriate pressure to the pocket. So far, we did not find any potential complication and the novel compression device were applicable to the patients in present trial. Besides, a more sophisticated compression device with measurement of pressure value is needed in the future. At present, no pressure-adjustable compression device is commercially available. Third, the proposed device is made of cotton fabric and medical silica gel, so it unlikely causes skin erosion, which is especially considerable for the elderly who have sensitive and delicate skin.

Despite these advantages, several limitations should be considered. First, all the participants were recruited from a single center, so our findings might not be generalizable to other populations. Second, the endpoint events regarding the assessment of pocket hematomas and skin erosions were subjective because they relied on the observers' judgment. Nevertheless, these discrepancies were minimized by adopting the standard pocket hematoma definition, and all the events were adjudicated by two physicians (26). Furthermore, the control groups were significantly older than the experimental group, but this fact did not seem to influence the observed incidence of hematoma. Lastly, the incidence of pocket hematomas was analyzed at the time of hospital discharge only. As such, potential bias in the interpretation of the results might occur.

## Conclusions

The incidence of pocket hematomas and skin erosions significantly decreases when the proposed pocket compression device is used for patients undergoing CIED implantation with uninterrupted DOACs. Thus, the length of hospital stay and re-operation rate can be decreased, and patient comfort can be improved. Further large multicenter studies should be performed to validate the effectiveness of the novel compression device in this population.

## Data Availability Statement

The raw data supporting the conclusions of this article will be made available by the authors, without undue reservation.

## Ethics Statement

The studies involving human participants were reviewed and approved by Human Ethics Research Committee of the Affiliated Hospital of Jiaxing University. The patients/participants provided their written informed consent to participate in this study.

## Author Contributions

Y-PF: conceptualization, methodology, software, investigation, and writing—original draft. LW: data curation, formal analysis, and writing—original draft preparation. C-YZ: resourses, visualization, and investigation. J-CS: software. H-LH: formal analysis, validation, and resourses. C-LZ: project administration and writing—reviewing and editing. C-JH: conceptualization, methodology, writing—reviewing and editing, and supervision. All authors contributed to the article and approved the submitted version.

## Funding

This research was funded by Jiaxing Science and Technology Program under Grant Nos. (2021AD30148, 2021AD30132, and 2020AY30006), Provincial-Municipal Joint Construction of Key Medical Disciplines in Zhejiang Province (2019-ss-xxgbx), Zhejiang Provincial Health Science and Technology Program under Grant Nos. (2022ZH011, 2021KY1105), Pioneer Innovation Team of Jiaxing Arteriosclerotic Diseases Research Institute (XFCX–DMYH), Jiaxing Institute of Arteriosclerotic Diseases (2020-dmzdsys), and Peak Discipline Established by the First Hospital of Jiaxing (GFXK-XXGNK).

## Conflict of Interest

The authors declare that the research was conducted in the absence of any commercial or financial relationships that could be construed as a potential conflict of interest.

## Publisher's Note

All claims expressed in this article are solely those of the authors and do not necessarily represent those of their affiliated organizations, or those of the publisher, the editors and the reviewers. Any product that may be evaluated in this article, or claim that may be made by its manufacturer, is not guaranteed or endorsed by the publisher.
